# Rat retinal vasomotion assessed by laser speckle imaging

**DOI:** 10.1371/journal.pone.0173805

**Published:** 2017-03-24

**Authors:** Anastasiia Y. Neganova, Dmitry D. Postnov, Olga Sosnovtseva, Jens Christian B. Jacobsen

**Affiliations:** Department of Biomedical Sciences, University of Copenhagen, Blegdamsvej 3, 2200 Copenhagen N, Denmark; Justus Liebig Universitat Giessen, GERMANY

## Abstract

Vasomotion is spontaneous or induced rhythmic changes in vascular tone or vessel diameter that lead to rhythmic changes in flow. While the vascular research community debates the physiological and pathophysiological consequence of vasomotion, there is a great need for experimental techniques that can address the role and dynamical properties of vasomotion *in vivo*. We apply laser speckle imaging to study spontaneous and drug induced vasomotion in retinal network of anesthetized rats. The results reveal a wide variety of dynamical patterns. Wavelet-based analysis shows that (i) spontaneous vasomotion occurs in anesthetized animals and (ii) vasomotion can be initiated by systemic administration of the thromboxane analogue U-46619 and the nitric-oxide donor S-nitroso-acetylDL-penicillamine (SNAP). Although these drugs activate different cellular pathways responsible for vasomotion, our approach can track the dynamical changes they cause.

## Introduction

It is known that disturbances in normal retinal blood flow are associated with various eye diseases, such as diabetic retinopathy and glaucoma [1–3]. Usually supply of a tissue with blood is controlled by various autoregulatory mechanisms, such as the myogenic response, conducted vasodilation, flow induced dilation etc. Vasomotion is oscillatory changes in the vascular tone which are accompanied by oscillatory changes in membrane potential [4, 5] and intracellular *Ca*^2+^ concentration [5, 6]. Vasomotion can be spontaneous or induced. Whereas the role of the myogenic response, conducted vasodilation and flow induced vasodilation are well understood, vasomotion remains a subject of intense study both by means of mathematical modeling and by means of animal experiments. It has been shown that the myogenic response [7] and vasomotion [8, 9] are involved in the control of the retinal blood flow. Depending on vessel size, specific vascular bed and species vasomotion frequencies are typically somewhere in the range of 3-20 cycles per minute [10]. Mayer waves is another phenomenon when delay in baroreceptor and chemoreceptor control systems causes slow oscillations in arterial blood pressure. In humans the frequency is around 0.1 Hz whereas in rats it is about 0.4 Hz [11, 12].

A number of studies have suggested that impaired vasomotion plays a distinctive role in the pathogenesis of different diseases such as chronic venous insufficiency [13], diabetes mellitus [14] and in eye diseases such as diabetic retinopathy [15] and maculopathy [3]. Very few experimental studies have, however, focused on vasomotion and its properties in the intact eye. In most cases these experiments have been performed in *in vitro* preparations of a single isolated vessel [8, 9] or a few vessels in a piece of tissue [5]. Most likely, this is due to the fact that vasomotion typically disappears in anesthetized animals, even if it was observed in awake animals [16, 17]. However, there are studies where vasomotion has been observed also in anesthetized animals [18]. In some cases only spontaneous vasomotion was subject to study [5]. In other cases both spontaneous vasomotion and vasomotion induced by chemicals such as U-46619, vasopressin, L-NAME or potassium were observed [8, 9]. To our knowledge there are no *in vivo* studies of vasomotion in rat retinal networks.

Laser speckle imaging (LSI) has been applied to study blood flow in various tissues [19–23]. This technique is non-scanning, full-field with high temporal and spatial resolution which makes it very attractive for various applications. Information about blood flow dynamics obtained by means of LSI is relative and absolute measurements require additional calibration as in Ref. [24]. LSI was initially developed to monitor blood flow in the retina [25]. It was used to study the changes in retinal blood flow in response to stimulation of photoreceptors with flickering light [26]. Flammer et al. [27] explored the correlation between impaired ocular blood flow and glaucoma, while Watanabe et al. [28] found that LSI technique is preferable to study choroidal blood flow in comparison with indocyanine green angiography. Ponticorvo et al. performed LSI via en endoscope to record blood flow images from the rat retina [29]. In our earlier work [30] we applied LSI to estimate diameter changes and blood flow responses upon administration of acetylcholine, angiotensin II and changing levels of anesthesia.

Here we present an experimental approach to detect and analyze spontaneous and induced vasomotion in the retinal vascular network *in vivo* by means of laser speckle imaging. We investigate dynamical properties of the response of multiple vessels to systemic administration of the thromboxane analogue U-46619 and the nitric-oxide donor S-nitroso-acetylDL-penicillamine (SNAP).

## Materials and methods

### Experiment

Experiments were carried out on male Sprague-Dawley rats with body weight 270-330 g, from Taconic (Lille Skensved, Denmark). Total number of rats used in the study was eight. The experimental protocol was approved by the Danish National Animal Experiments Inspectorate and were conducted in accordance with guidelines of the American Physiological Society.

An IR Laser Module (LDM785, 785 nm, 20 mW, Thorlabs, Newton, New Jersey) was used as laser beam source. To deliver the laser beam to the rat eye an endoscope (5 mm in diameter, 11.5 cm in length, Karl Storz 67260AA, Tuttlingen, Germany) was used [29]. The images were captured by a CMOS camera (acA1300-60gmNIR, Basler, Ahrensburg, Germany) with the following settings: recording rate of 50 frames per second, 800 × 800 pixel resolution, and 10 ms exposure time.

Before surgery the rat was anesthetized with 5% isofluorane, and then during the surgery anesthetic concentration was reduced to 2%. Two polyethylene catheters were inserted into the left jugular vein to administrate chemicals. One catheter was placed in the right carotid artery and connected to a pressure transducer (Statham P23-dB, Gould, Oxnard, CA) for continuous measurements of the blood pressure. A tracheotomy was performed and the anesthetic gas was hereafter delivered through the tube inserted into the trachea. After the surgery the rat was placed on a heating table, where all experiments were carried out. Mechanical ventilation of the lungs was performed by a small-animal respirator (60 breaths/min). To prevent eye movements the rat was paralyzed with continuous I.V. infusion of gallamine triethiodide in a concentration of 0.33 mg/kg in saline solution at the rate of 20 *μ*l/min. Before the experiment atropine was applied on the eye surface to dilate the pupil. The rat was euthanized at the end of an experiment by decapitation under anesthesia with 3% isoflurane. Full description of the experimental procedure is given in Ref. [30].

**Fig 1:**
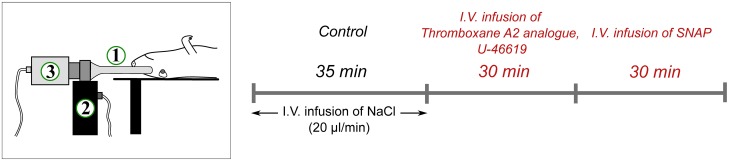
Schematic presentation. The experimental set up (left panel) and the experimental protocol (right panel). Experimental setup includes endoscope (1), laser module (2), and CMOS camera (3). The rat was anesthetized and paralyzed. Experimental protocol includes I.V. infusion of U-46619 and SNAP.

The experimental protocol included an initial 35 min control period with saline solution (instead of drug) infused at the rate of 20 *μ*l/min. This period was then used to detect spontaneous vasomotion and as the baseline reference to estimate the drug response. The control period was followed by 30 min systemic (intravenous) infusion of U-46619 in a concentration of 1 *μ*g/(kg⋅min) and further continuous intravenous infusion of SNAP in a concentration of 85 nmol/(kg⋅min) at the rate of 20 *μ*l/min. The time line illustrating the experimental procedure is presented in Fig 1.

We did not know a priory if spontaneous vasomotion is sufficiently prominent to be detected in the subsequent data analysis (if too few vessels show activity or if the amplitude of the oscillations is too small). The resting period may therefore not tell us whether the method is, in general, applicable to detect this kind of oscillations in the retinal vascular bed. Therefore, we aimed at subsequently stimulating the vascular bed to strengthen preexisting oscillations or push the vessels into an oscillatory domain:

- Since U-46619 is known to induce vasomotion in porcine retinal arterioles [8, 9] we found this drug to be a reasonable initial choice;
- To further enhance the chance of observing oscillations we added a period with SNAP, a nitric oxide donor, known to induce vasomotion elsewhere (particularly well investigated in the mesenteric bed) and whose effect on vasomotion seems to be contradictory [6, 31, 32];
- We are aware that U46619 is a stable thromboxane analog with residual activity likely to last throughout the NO infusion period [33] but this is not in conflict with the prime aim of enhancement of oscillations.

### Analysis

Recorded raw laser speckle data were processed using spatial laser speckle contrast analysis to obtain speckle blood flow dynamics [34]:

$$\begin{array}{c} SV={\bar{I}}^{2}/\sigma^{2}, \end{array}$$ (1)

where $\bar{I}$ and *σ* are the mean value and standard deviation for speckle intensities calculated over 25 pixels in a 5 × 5 window. The moving window covered the whole image with the step of 1 pixel. To validate rhythmic activity detected in the *SV* data we compared blood pressure variations with the activity observed in the *SV*(*x*, *y*) data set of 800 × 800 pixels recorded with the rate of 50 frames per second. Since analysis of such data sets is very demanding from a computational point of view (more than 300 Gb per data set), we down sampled the data by averaging 25 consequent frames. This resulted in *SV*(*x*, *y*) data set of 800 × 800 pixels with the rate of 2 frames per second which is enough to satisfy Nyquist criteria for vasomotion and breathing signals.

**Fig 2:**
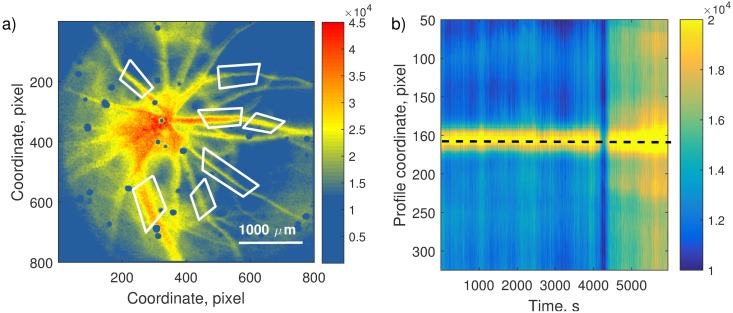
Image segmentation. A representative example of image segmentation. a) Mean SV frame. Red polygons mark regions for vessel segmentation. b) Vessel *SV* profile as a function of time. Values at each moment of time are calculated as averaged values along scan lines perpendicular to the vessel axis over the selected region. The center line (dashed) always belongs to the individual vessel (high *SV* values).

To extract *SV* along individual vessels and to remove motion artifacts we applied a segmentation algorithm described in Ref. [35]. Several regions of interests with well-defined vessels were selected as shown in Fig 2(a). The segmentation algorithm masked the vessel in the selected region and determined artifact-free *SV* values along the center line of the vessel for all frames (Fig 2(b)).

The rhythmic activity we are interested in has time-dependent properties. Analysis, therefore, requires a method that can determine frequency as a function of time. We used wavelet transform to satisfy this need and to provide better frequency resolution [36]. The wavelet transform of a signal x(t) can be written as:

$$\begin{array}{c} W_{x}\left(a,b\right)=\frac{1}{\sqrt{a}}\int_{-\infty }^{\infty }x\left(t\right)\psi_{a,b}^{*}\left(t\right)dt, \end{array}$$ (2)

where *ψ* is referred to as the “mother” function with *a* and *b* characterizing the timescale and temporal localization, respectively; the asterisk refers to the complex conjugate; and *x*(*t*) is the time series. For this analysis we used the continuous Morlet transform from the MATLAB toolbox, with corresponding conversion of scale *a* to frequency *f*. From *W*_*x*_(*f*, *b*) the time-dependent wavelet energy *E*_*x*_(*f*, *b*)∼∣*W*_*x*_(*f*, *b*)∣^2^ was estimated. Wavelet energy was calculated for 81 vessel over 8 animals. To display wavelet energy spectra, the energy was averaged over 10 minutes for each step of the protocol (control, U-46619, and SNAP) starting 10 minutes after each infusion.

To analyze and quantify periodic activity in the averaged wavelet spectra we identified peaks in 243 spectra across 81 vessels. For each spectrum (i) the peak frequency *f* was identified as the frequency corresponding to the local maximum of the most prominent peak in the frequency band of interest and (ii) the peak prominence as the peak height compared to the noise background. For further analysis we suggest several characteristics.

**Fig 3:**
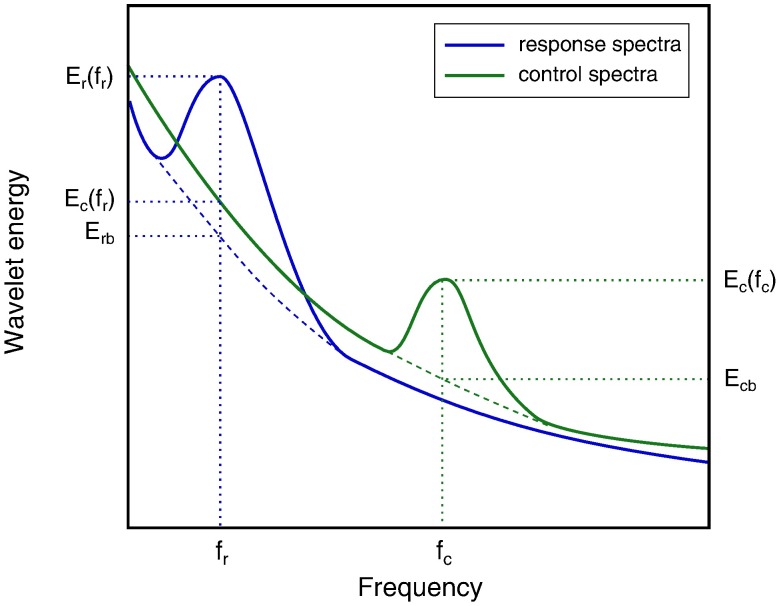
Schematic presentation of characteristics used for spectral analysis. Blue curve is the response (U-46619 or SNAP) wavelet spectrum, green curve is the control spectrum. *f*_*r*_ and *f*_*c*_ are the frequencies of the most prominent peaks in the response and control spectra, respectively. *E*_*r*_(*f*_*r*_) and *E*_*c*_(*f*_*r*_) are the energy values in the response and control spectra, respectively. *E*_*rb*_ is the energy value of the noise background in the response spectrum at *f*_*r*_, and *E*_*cb*_ is the energy value of the noise background in the control spectrum at *f*_*c*_.

Energy ratio:

$$\begin{array}{c} E=\frac{E_{r}\left(f_{r}\right)}{E_{c}\left(f_{r}\right)}, \end{array}$$ (3)

where *E*_*r*_(*f*_*r*_) and *E*_*c*_(*f*_*r*_) are energy values in response and control spectra, respectively, at the frequency of the most prominent peak (*f*_*r*_) in the response spectrum (see Fig 3). This characteristic describes the relative change in energy at the frequency of the most prominent peak in the response spectrum (during U-46619 or SNAP infusions) compared to the control spectrum.

Prominence ratio:

$$\begin{array}{c} P=\frac{P_{r}}{P_{c}}. \end{array}$$ (4)

Here *P*_*r*_ = *E*_*r*_(*f*_*r*_)−*E*_*rb*_ and *P*_*c*_ = *E*_*c*_(*f*_*c*_)−*E*_*cb*_. *E*_*rb*_ and *E*_*cb*_ are energy values of the noise background in the response and control spectra, respectively (Fig 3). This ratio describes whether the most prominent peak in the response *P*_*r*_ is more prominent than the most prominent peak in the control *P*_*c*_ for a selected frequency band. This characteristic indicates whether there is an increase or decrease of the activity at the chosen frequency. With this characteristic we calculated the number of vessels *n* in each animal for which *P* > 1.1, i.e. these vessels demonstrated at least 10% increase in activity during drug infusion as compared to the control recordings. Frequencies of induced vasomotion were compared for two different drug administrations by means of paired t-test. Significant difference corresponds to p-values < 0.05.

## Results

### Validation of rhythmic activity

To validate activity detected in *SV* data we compared wavelet spectra of continuously recorded blood pressure with the spectra obtained by laser speckle imaging at the rate of 50 frames per second. The measurements were performed on the same animal.

**Fig 4:**
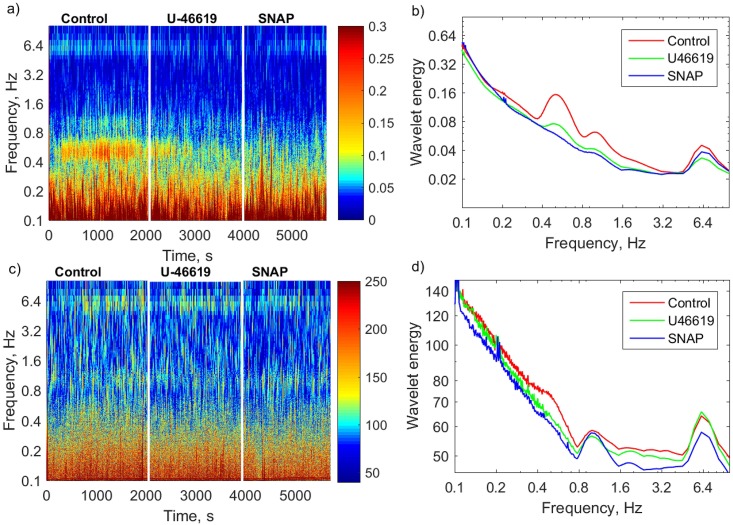
Wavelet analysis of arterial pressure and *SV* recordings. Analysis of (a,b) arterial blood pressure and (c,d) *SV* of a single retinal vessel of a representative rat. Left panels: Color-coded wavelet energy over frequencies and time. Three time intervals are selected according to the experimental protocol: control, U-46619, and SNAP. Right panels: Wavelet energy spectra. Different colors code different time intervals as the legend describes. The peak at 0.5 Hz corresponds to Mayer waves, 1 Hz corresponds to ventilation frequency and 6.4 Hz corresponds to the pulse. *SV* analysis reveals the same frequencies although shape of the peaks and response to drug infusion are different.

Wavelet analysis of arterial blood pressure is presented in Fig 4(a) and 4(b). Color-coded figure (a) shows the distribution of wavelet energy over frequencies and time. White lines divide the time line into control (35 minutes), U-46619 administration period (30 min) and SNAP administration period (30 min). Three peaks are well distinguished in the wavelet spectrum during control period (Fig 4(b), red curve):

- The peak around 6.4 Hz is the frequency of the heart beat. This rhythm is well pronounced during all periods of the experiment as depicted in Fig 4(a);
- The peak around 1 Hz is the frequency of lung ventilation. The influence from ventilation is somewhat reduced during drug infusion (Fig 4(b), green and blue spectra);
- The oscillations with the frequency of 0.4–0.5 Hz most likely correspond to Mayer waves [11, 12].

Wavelet analysis of *SV* data calculated with high sampling rate (50 frames per second) is shown in Fig 4(c) and 4(d). One can distinguish peaks at the same frequencies (d) as in the spectra for the arterial blood pressure (b), although the degree of activity in the control and response periods is different. The vessel in Fig 4(d) does not show independent vasomotor activity. Since the same pattern is observed both in blood pressure recordings and *SV* data, this ensures that *SV* analysis reliably detects oscillatory activity. Differences in peak shape and response to drugs highlight the fact that local retinal blood flow dynamics is different compared to blood pressure changes in carotid artery and can be caused by nonlinear relation between blood flow dynamics and blood pressure.

### Detection of vasomotion activity

**Fig 5:**
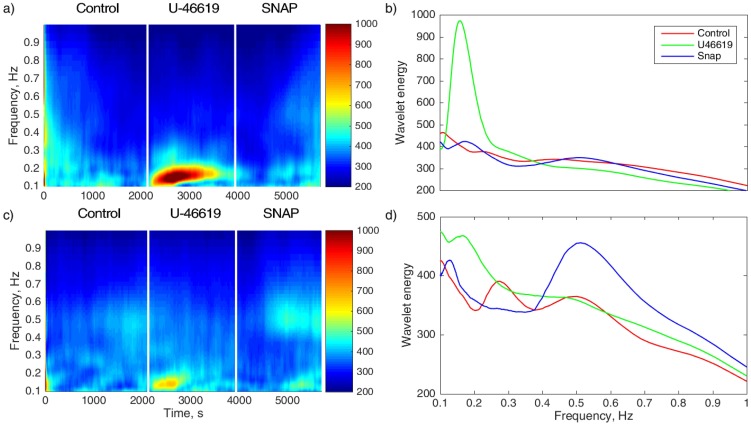
Wavelet analysis of *SV* recordings. Response of two vessels in the same representative rat. Left panels: Color-coded wavelet energy over frequencies and time. Right panels: Wavelet energy spectra where different colors correspond to control (red), U-46619 administration (green) and SNAP administration (blue) periods. Note that vessels in the same network may respond differently: (a,b) One vessel demonstrates vasomotion activity at the frequency of about 0.17 Hz in response to infusion of U-46619 and no Mayer wave activity is observed. (c,d) The other vessel demonstrates a weaker response to U-46619 at about 0.15 Hz but shows Mayer wave activity at 0.5 Hz in response to SNAP administration.

Fig 5 represents wavelet analysis of *SV* data for two different vessels from the same animal. Data were collected and analyzed during control period, U-46619 administration and SNAP administration. The left panels show wavelet energy distributed over frequencies and time. The two vessels react differently upon administration of the same drug. One can clearly see bright spots during U-46619 administration in two vessels (Fig 5(a) and 5(c)) but the activity in the first vessel (a) is almost twice as strong and longer lasting. On the other hand, the second vessel (c) shows prolonged activity at a different frequency during SNAP administration. Spectral analysis (right panels) provides details of frequency properties of the observed rhythmic activities. The first vessel (b) does not show spontaneous activity in the control period (red) but responses strongly to U-46619 infusion (green) at a frequency of about 0.17 Hz. This might correspond to induced vasomotion. The second vessel (d) shows enhanced response to SNAP administration (blue) at a frequency of around 0.5 Hz possibly corresponding to Mayer waves. Although these figures are used as an example of possible responses, they illustrate to which extent the laser speckle imaging together with wavelet analysis can detect a variety of dynamical patterns.

To reveal common patterns of dynamical behavior we segmented and analyzed 81 vessels from 8 independent experiments. The Table 1 summarizes information about frequency *f* and relative energy *E* of the detected rhythmic activity, as well as statistical data about number of vessels *n* and *m* demonstrating induced and spontaneous vasomotion, respectively. We compared the effect of U-46619 and SNAP on flow dynamics within two frequency bands (0.1–0.3 Hz and 0.3–0.7 Hz) where one would expect to find retinal vasomotion [8, 9] and Mayer waves [11, 12].

**Table 1:** Change of *SV* dynamics in response to drug infusion. “Exp” denotes experiment (animal) number. *N* is the number of vessels segmented from individual experiments. *m* is the number of vessels for which spontaneous oscillatory activity (at least 10% above the noise background) was detected. *E* is the wavelet energy of the most prominent peak in the response relative to the wavelet energy at the same frequency in the control (Eq 3). *f* is the most prominent frequency in the corresponding frequency band. Energies and frequencies correspond to the vessels with induced vasomotion, i.e. to *n*_1_ and *n*_2_ vessels. Indexes 1 and 2 denote frequency bands of 0.1–0.3 Hz and 0.3–0.7 Hz, respectively. *n* is the number of vessels for which prominence of the highest peak (Eq 4) in the response is at least 10% higher than in the control.

U-46619 administration									
Exp	N	*m*_1_	*E*_1_	*f*_1_, Hz	*n*_1_	*m*_2_	*E*_2_	*f*_2_, Hz	*n*_2_
1	8	2	1.85±0.99	0.20±0.05	6	0	1.46±0.62	0.42±0.05	6
2	9	0	0.94±0.37	0.16±0.04	3	7	0.76±0.30	0.47±0.07	0
3	9	0	0.93±0.16	0.18±0.05	3	5	0.96±0.09	0.46±0.07	3
4	11	0	1.05±0.19	0.19±0.04	9	0	1.00±0.21	0.48±0.12	5
5	11	3	1.06±0.15	0.15±0.05	4	9	1.00±0.11	0.51±0.01	6
6	11	2	0.94±0.10	0.19±0.06	5	1	0.97±0.10	0.46±0.09	3
7	11	0	1.52±0.74	0.17±0.04	9	2	1.43±0.79	0.45±0.04	2
8	11	1	1.73±0.78	0.15±0.02	10	0	1.50±0.82	0.41±0.05	5
Total	81	8	1.25±0.42	0.17±0.04	49	24	1.13±0.38	0.46±0.06	30
SNAP administration									
1	8	2	1.62±0.87	0.22±0.03	7	0	1.28±0.51	0.45±0.08	8
2	9	0	1.31±0.94	0.19±0.06	6	7	1.07±0.52	0.46±0.08	0
3	9	0	1.02±0.33	0.16±0.05	8	5	1.07±0.18	0.50±0.03	5
4	11	0	1.16±0.37	0.16±0.05	6	0	1.23±0.30	0.38±0.07	2
5	11	3	1.02±0.33	0.17±0.04	6	9	0.87±0.34	0.45±0.06	2
6	11	2	1.02±0.28	0.14±0.03	4	1	1.13±0.17	0.49±0.07	10
7	11	0	1.12±0.15	0.17±0.05	7	2	1.14±0.12	0.46±0.07	8
8	11	1	1.36±2.12	0.15±0.06	4	0	1.47±2.51	0.45±0.08	8
Total	81	8	1.19±0.67	0.17±0.05	48	24	1.16±0.60	0.45±0.07	43

**Fig 6:**
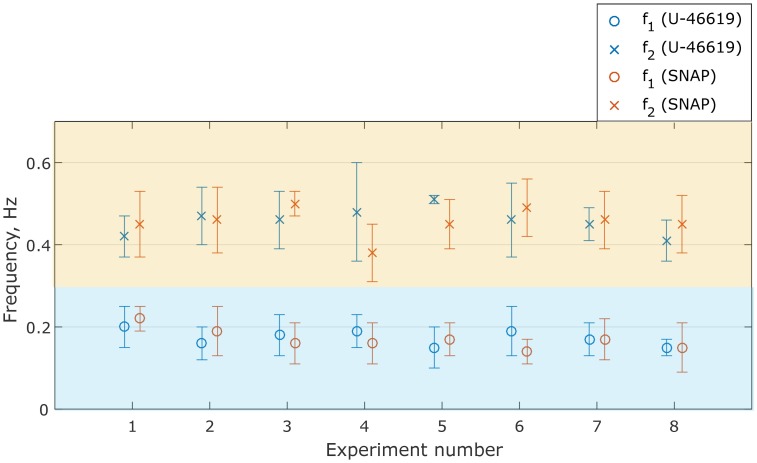
Distribution of frequencies. Frequencies of the most prominent peaks within the frequency band of 0.1–0.3 Hz (circle) and 0.3–0.7 Hz (cross) are the same for U-46619 (blue) and SNAP (orange) time series. Paired t-test indicates that frequencies of vasomotion induced by administration of SNAP and U-46619 do not show significant difference (p-values are 0.7 and 0.9 for two frequency bands). Vertical lines represent standard deviation. Light orange and light blue regions mark two selected frequency bands.

**Fig 7:**
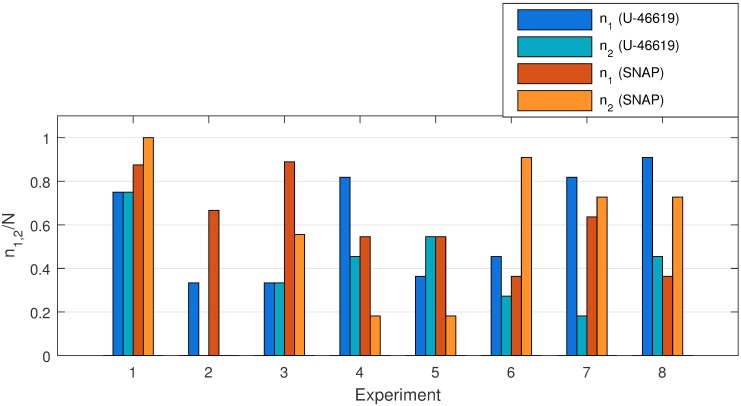
Distribution of number of vessels with increased activity. The ratio of number of vessels *n*_1,2_ with increased activity in response to drug administration to the total number of vessels *N* allocated in an individual experiment. The activity is estimated in two frequency bands: 0.1–0.3 Hz (dark blue and dark orange) and 0.3–0.7 Hz (light blue and light orange).

To quantify changes in oscillatory activity we introduced a few characteristics:

- *m* is the number of vessels for which spontaneous oscillatory activity during control was detected. Notice that spontaneous activity considered to be detected only if the peak energy was at least 10% above the noise background, i.e. *E*_*c*_(*f*_*c*_)/*E*_*cb*_ > 1.1 (see Fig 3);
- *E* describes a change of wavelet energy (i.e, the height of the most prominent peak in the wavelet spectrum) in drug response measurements relatively to control measurements (Eq 3). In other words, *E* shows the change in oscillatory activity within a certain frequency band after drug administration. *E* values more than 1.0 represent increasing activity while *E* values less than 1.0 indicate decreasing oscillatory activity in response to drug administration. Inspection of columns *E*_1_ and *E*_2_ reveals that administration of U-46619 and SNAP leads to an increase of averaged oscillatory activity in both frequency bands (0.1–0.3 Hz and 0.3–0.7 Hz);
- *f* is the frequency of the most prominent peak in the corresponding frequency range. One can see that the administration of U-46619 and SNAP increases oscillatory activity at similar frequencies of 0.17 Hz and 0.45–0.46 Hz, respectively. This is visualized in Fig 6. Notice that while there is some variability for different vessels in each animal, the mean frequency values are close for both drugs. This possibly indicates that while two drugs affect the vasculature via different signaling pathways, vasomotion characteristics are something vessel specific, independent of the eliciting stimulus;
- *n* is the number of vessels for which the prominence of the peak in the response is higher than in the control (*P* > 1.1, Eq 4), i.e. vessels show induced vasomotion. The results are visualized in Fig 7. It presents the fraction of vessels that demonstrate increasing activity within two selected frequency ranges for U-46619 and SMAP administration. The experiments show that more vessels in the rat retina demonstrate pronounced oscillatory activity in response to U-46619 administration in the lower frequency range (dark blue) while SNAP administration initiates activity in both frequency ranges (orange). We performed additional analysis for *n*_1_ and *n*_2_ by defining the threshold for the peak energy being at least 10% above the noise background (*E*_*r*_(*f*_*r*_)/*E*_*rb*_ > 1.1). The analysis showed that after U-46619 administration 63% of 49 vessels (*n*_1_) and 67% of 30 vessels (*n*_2_) demonstrated activity above the threshold. U-46619-induced activity (i.e., activity that was not detected during the control period but was initiated by drug infusion) above the threshold was detected in 26 vessels and in 12 vessels for two frequency bands. After SNAP administration 40% out of 48 vessels (*n*_1_) and 47% out of 43 vessels (*n*_2_) demonstrated an enhanced activity above the threshold but only 16 and 15 vessels, respectively, demonstrated SNAP-induced activity.

## Conclusion

There is no full understanding how vasomotion appears and what its role is in the development of pathological conditions. Such involvement was investigated in different vascular beds in *in vivo* and *in vitro* but results are contradictory [6, 32]. Although spontaneous vasomotion has been observed in human subjects, it is reported to be suppressed under anesthesia which complicates *in vivo* studies [16, 17]. The easy access to retinal vasculature provides an opportunity for non-invasive medical diagnostics.

To assess vasomotion *in vivo* we applied laser speckle imaging to monitor relative flow changes in retinal vascular network and wavelet analysis to reveal features of the rhythmic activity. We provided simultaneous continuous monitoring of arterial pressure and blood dynamics recorded by means of laser speckle imaging to ensure local origin of the observed flow changes. *SV* data demonstrated the same systemic activity (6.4 Hz rate of heart beat and 1 Hz lung ventilation) as the blood pressure measurements.

Significant (at least 10% above the noise background) spontaneous activity was observed in 8 vessels out of 81 in the frequency range of 0.1–0.3 Hz (vasomotion) and in 24 vessels in the frequency range of 0.3–0.7 Hz (Mayer waves). Moreover, analysis of induced vasomotion showed that both U-46619 and SNAP initiated activity at 0.17 Hz and 0.45–0.46 Hz frequencies. Interestingly, the oscillations at frequencies 0.45–0.46 Hz in the speckle blood flow dynamics data appeared or became more pronounced after the administration of SNAP and to a lesser degree after the administration of U-46619.

The results indicate the important applicability of laser speckle imaging as a tool to address open questions of *in vivo* studies about dynamical features of retinal vascular networks and appearance of vasomotion. Combined with vessel diameter estimation [30, 35] such approach can become a powerful tool to study vasomotion associated with pathological conditions.
